# Numerical and Experimental Verification of a Multiple-Variable Spatiotemporal Regression Model for Grout Defect Identification in a Precast Structure

**DOI:** 10.3390/s20113264

**Published:** 2020-06-08

**Authors:** Xuan Zhang, Hesheng Tang, Deyuan Zhou, Shanshan Chen, Taotao Zhao, Songtao Xue

**Affiliations:** Department of Disaster Mitigation for Structures, Tongji University, Shanghai 200092, China; muzixuan@tongji.edu.cn (X.Z.); 85032@tongji.edu.cn (D.Z.); 1530735@tongji.edu.cn (S.C.); 1732344@tongji.edu.cn (T.Z.); xue@tongji.edu.cn (S.X.)

**Keywords:** local defect identification, linear regression model, damage indicator, beam–column connection, concrete frame structure

## Abstract

Due to the increased service life, environmental corrosion, unreasonable construction, and other issues, local defects inevitably exist in civil structures, which affect the structural performance and can lead to structural failure. However, research on grout defect identification of precast reinforced concrete frame structures with rebars spliced by sleeves faces great challenges owing to the complexity of the problem. This study presents a multiple-variable spatiotemporal regression model algorithm to identify local defects based on structural vibration responses collected using a sensor network. First, numerical simulations were carried out on precast beam–column connection models by comparing the identification results based on a single-variable regression model, two-variable spatial regression model, and two-variable spatiotemporal regression model; furthermore, a multiple-variable spatiotemporal regression model was proposed and robustness analysis of the damage indicator was carried out. Then, to explore the validity of the proposed method, a nondestructive vibration experiment was considered on a half-scaled, two-floor, precast concrete frame structure with column rebars spliced by defective grout sleeves. The results show that local defects were successfully identified based on a multiple-variable spatiotemporal regression model.

## 1. Introduction

Due to overloading, environmental corrosion, material aging, operating loads, fatigue, and other unexpected events, engineering structures inevitably sustain different kinds of defects that can lead to structural failure. Therefore, it is necessary to develop a defect identification method to guarantee structural safety [1,2,3,4,5]. 

Structural physical parameters will be altered due to structural damage, which will result in a difference in the corresponding dynamic characteristics (e.g., natural frequency, mode curvature, and strain mode), through which structural damage can be identified [6,7,8,9]. Several studies have focused on damage identification based on structural global responses. Based on natural frequency responses, Sha et al. [10,11,12] carried out damage detection in beams, aluminum samples, and a soil box according to numerical simulations and experiments. The results demonstrated the capability of the methods to localize damage and to estimate the damage severity. Based on modal flexibility, the procedure used to detect and locate damage was experimentally validated and applied to a simply supported beam, a long-span arch bridge, a full-scale long span, and circular-hollow-sectional and box-sectional steel cantilever beams by Su et al. [13,14,15], in which damage was identified. Cui et al. [16] defined a damage-detection method according to strain modes under ambient excitation, and experiments on single-damage and multi-damage cases with different degrees and locations were performed, in which damage cases were reasonably detected. Damage identification based on structural local vibration responses has also been proposed. Dorvash et al. [17,18] proposed local damage-detection algorithms based on the regression of vibration responses and they validated their performance on a beam–column connection and a frame structure, where both algorithms were shown to be effective. A local-damage identification method was proposed by Naito [19], and a local vibration test was conducted on a portion of a deteriorated concrete deck with results indicating that the method can be applied to detect damage in concrete decks. Downey [20] proposed a dense sensor network to derive a data-driven damage-detection and localization method for wind turbine blades, where numerical simulations demonstrated the method’s ability to distinguish healthy sections.

Recently, precast structures have attracted much attention. In a precast concrete structure, sleeve grouting of rebar connections is widely used throughout the world. However, sleeve grout defects always exist, such as sleeve-end defects caused by the leakage of slurry, middle defects caused by incompletely discharging air, and the eccentric defects of steel [21]. Therefore, several studies focused on grout defect identification based on different methods. Li et al. [22] established a piezoelectric impedance model for a prefabricated structure, where grouting defects were identified qualitatively using a statistical method showing that piezoelectric impedance analysis can effectively identify defects in the structure nodes. By comparing the actual experimental results and the simulation parameter model, a method for detecting the fullness of a grouting sleeve based on microwave radio frequency S parameters was proposed by Zheng et al. [23], which provided a valuable reference for the actual engineering inspection. Based on the differences between the guided wave signals of a healthy specimen and defective specimens, Li et al. [24] explored the influence of the grouting defects on the guided wave signal through experiments and simulations, where the results showed that there was a positive correlation between the proposed damage index and grouting defects. A preformed-aisle device was designed to inspect the grouting fullness of a sleeve of prefabricated structures by Sun et al. [25]. The results showed that the construction defects of insufficient grouting caused by grout leakage were located at the top of the sleeve and the device was effective at checking the grouting fullness at the outlet port of the sleeve. Based on the X-ray digital radiography method, grout inspection on precast shear wall specimens and precast sandwich insulation shear wall specimens was conducted by Li. et al. [26], where the results indicated that the technology was feasible for grouting quality inspection for single-row-centered walls.

In summary, there have been several studies on grout defect identification. However, these methods have limits due to the expensive testing equipment, complex operation, or destructive detection. The nondestructive identification methods based on regression models have the advantage of not needing to know the structural characteristics in advance. Furthermore, the measuring point arrangement is easy and flexible, and sensors need only be arranged near the component of concern. As there are few studies on sleeve grout defect identification of precast concrete structures based on regression models via vibration responses, in this study, we adopted single-variable regression (SVR), two-variable spatial regression (TVSR), and two-variable spatiotemporal regression (TVSTR) model algorithms to identify grout defects in a precast beam–column connection model simulated in finite element software ABAQUS [27]. By analyzing and comparing the results of the three algorithms, a multiple-variable spatiotemporal regression model algorithm was proposed. The experiment was conducted on a half-scaled, two-floor, precast concrete frame structure with column rebars spliced by grout sleeves to explore the validity of the multiple-variable spatiotemporal regression model on local defect identification and good results were obtained.

## 2. Methodology

### 2.1. Theory Background

#### 2.1.1. Linear Regression Model

In this paper, the initial linear-elastic structure was assumed to remain linear-elastic after damage. Hence, a linear relationship was used to depict the structural nodes’ responses. A linear regression model was applied to depict this relationship:

(1) $$Y=\alpha +\sum \beta_{i}X_{i}+\varepsilon ,$$

where *Y* is the dependent variable, *X_i_* is the independent variable, *α* is the intercept, *β_i_* is the regression coefficient, and *ε* is the random error in regression prediction, which is mainly due to noise in the data acquisition and the neglected higher-order modal dynamic response.

Three linear regression models were adopted: SVR, TVSR, and TVSTR [28,29,30,31].

1. SVR model

The SVR model has only one independent variable and one dependent variable:

(2) $${\hat{u}}_{j}\left(t_{k}\right)=\alpha_{ij}u_{i}\left(t_{k}\right)+\varepsilon_{ij}\left(t_{k}\right)+\beta_{ij},$$

where *u_i_*(*t_k_*) is the independent variable, representing the vibration response data (such as displacement, velocity, and acceleration) of node *i* at moment *t_k_*; ${\hat{u}}_{j}\left(t_{k}\right)$ is the dependent variable, representing the estimated dynamic response data of node *j* at moment *t_k_*; *α_ij_* is the influence coefficient between node *i* and node *j* at moment *t_k_*, which changes with the variation of external excitation and structural stiffness; *ε_ij_*(*t_k_*) is the random error in the regression prediction; and *β_ij_* is the intercept.

2. TVSR model

The TVSR model has two independent variables and one dependent variable:

(3) $${\hat{u}}_{j}\left(t_{k}\right)=\alpha_{1j}u_{1}\left(t_{k}\right)+\alpha_{2j}u_{2}\left(t_{k}\right)+\varepsilon_{ij}\left(t_{k}\right)+\beta_{ij},$$

where *u_1_*(*t_k_*) and *u_2_*(*t_k_*) are the independent variables, *α_1j_* is the regression coefficient between node 1 and node *j*, and *α_2j_* is the regression coefficient between node 2 and node *j*. The vibration response data of the two independent variables come from the nodes near the dependent variable data node.

3. TVSTR model

The TVSTR model also has two independent variables and one dependent variable. However, different from the TVSR model, the dynamic response data of the two independent variables are from the same node and different moments:

(4) $${\hat{u}}_{j}\left(t_{k}\right)=\alpha_{ij}u_{i}\left(t_{k}\right)+\alpha_{ij}^{’}u_{i}\left(t_{k-1}\right)+\varepsilon_{ij}\left(t_{k}\right)+\beta_{ij},$$

where *α’_ij_* is the regression coefficient between nodes *j* and *i* at moment *t_k-1_*, and *u_i_*(*t_k-1_*) is the dynamic response data of node *i* at moment *t_k-1_*. The space and time constraints of the two independent variables are considered simultaneously.

#### 2.1.2. Accuracy Verification

To verify the fitting degree of the linear relationship, the coefficient of determination (CoD) *r^2^* was introduced in the SVR model, and the adjusted CoD *r^2^_adj_* was introduced to the TVSR and TVSTR models. These are calculated as follows:

(5) $$r^{2}=1-\frac{\sum_{i}{\left(u_{j}-{\hat{u}}_{j}\right)}^{2}}{\sum_{j}{\left(u_{j}-\bar{u}\right)}^{2}},$$

(6) $$\;r_{adj}^{2}=1-\frac{\sum_{j=1}^{n}\frac{{\left(u_{j}-{\hat{u}}_{j}\right)}^{2}}{\left(n-2\right)}}{\sum_{j=1}^{n}\frac{{\left(u_{j}-\hat{u}\right)}^{2}}{\left(n-1\right)}},$$

where $u_{j}$ is the actual value of node *j* and $\hat{u}$ is the average value of $u_{j}$. The fitting effect of the estimated regression model is better when *r^2^* and *r^2^_adj_* are closer to 1 and worse as they approach zero. Generally, if the value of *r^2^* or *r^2^_adj_* is smaller than 0.8, the fitting effect of the linear relationship is not good.

#### 2.1.3. Damage Indicator

The linear relationship between the nodes’ responses in a defective structure is different than in a non-defective structure, and this is reflected in the influence coefficients of the linear regression model. By comparing the influence coefficients of non-defective and defective states, the change of the linear relationship can be identified. In other words, by measuring the angle *θ* between the regression lines (or planes) of the two states, the damage location can be identified. Therefore, angle *θ* is defined as the damage indicator. 

**Figure 1:**
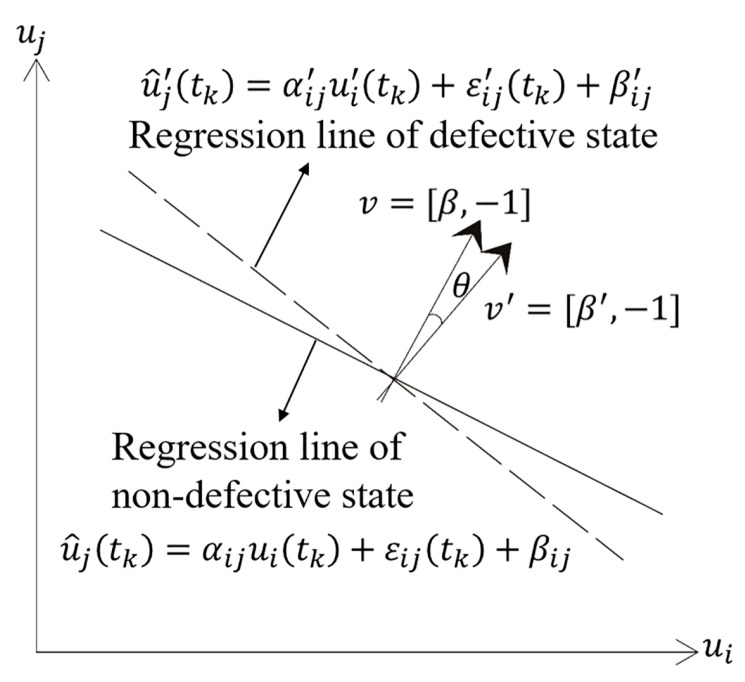
Geometric significance of the damage indicator *θ* in a single-variable regression (SVR) model.

The damage indicator θ in the SVR model is calculated as follows:

(7) $$\theta =\cos^{-1}\left|\frac{vv\prime }{\left\|v\|\|v\prime \right\|}\right|=\cos^{-1}\left|\frac{\beta \beta \prime +1}{\sqrt{\beta^{2}+1}\sqrt{\beta^{\prime 2}+1}}\right|$$

where *v =* [*β*, −1] is the normal vector of the regression line of the non-defective state and *v′ =* [*β′*, −1] is the normal vector of the regression line of the defective state. Figure 1 shows the geometric significance of *θ* in the SVR model.

**Figure 2:**
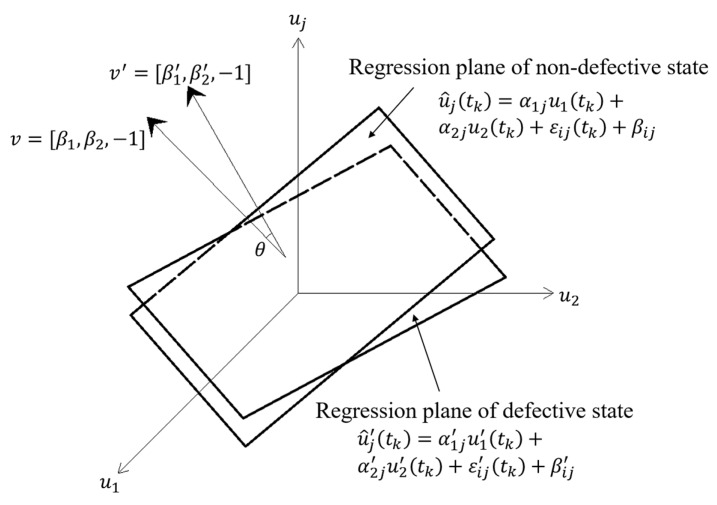
Geometric significance of the damage indicator *θ* in a two-variable spatial regression (TVSR) model.

**Figure 3:**
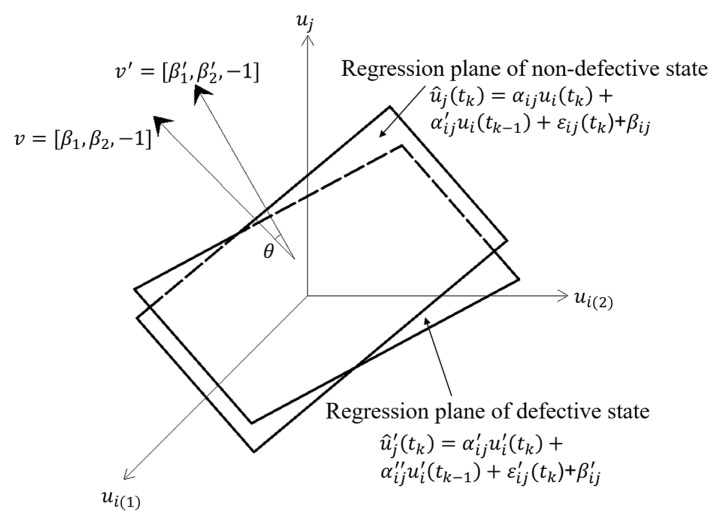
Geometric significance of the damage indicator *θ* in a two-variable spatiotemporal regression (TVSTR) model.

The damage indicator *θ* in the TVSR and TVSTR models is calculated as follows:

(8) $$\theta =\cos^{-1}\left|\frac{vv\prime }{\left\|v\|\|v\prime \right\|}\right|=\cos^{-1}\left|\frac{\beta_{1}\beta_{1}\prime +\beta_{2}\beta_{2}\prime +1}{\sqrt{\beta_{1}^{2}+\beta_{2}^{2}+1}\sqrt{\beta_{1}\prime^{2}+\beta_{2}\prime^{2}+1}}\right|,$$

where *v =* [*β_1_*, *β_2_*, −1] and *v′=* [*β_1_′*, *β_2_’*, −1]. Figure 2 and Figure 3 show the geometric significance of *θ* in the TVSR and TVSTR models, respectively.

### 2.2. Proposed Methodology

All steps used in this methodology are condensed as follows:

1. When investigating both the non-defective and defective structures, the acceleration responses of nodes near sleeves in both structures were obtained using an accelerometer network.
2. A reasonable linear regression model (SVR model, TVSR model, TVSTR model, or the proposed multiple-variable model proposed in Section 3.3.4) was adopted to analyze the obtained acceleration responses.
3. The coefficient of determination (CoD) *r^2^* or the adjusted CoD *r^2^_adj_* was calculated to verify the accuracy of the linear regression model.
4. Every damage indicator was calculated based on the two linear regression models of the non-defective and defective states. Generally, the damage indicators of nodes near defects were larger than those of other nodes, and the locations of the defects could be identified.

## 3. Numerical Simulation on Grout Defect Identification in Precast Beam-Column Connection

### 3.1. Finite Element Model (FEM)

To verify the performance of the proposed local damage detection method, we simulated a precast beam–column connection in ABAQUS finite element software, with longitudinal rebars spliced by a grout sleeve, and the sleeve grout defect was simulated via stiffness reduction of the grouting concrete.

**Figure 4:**
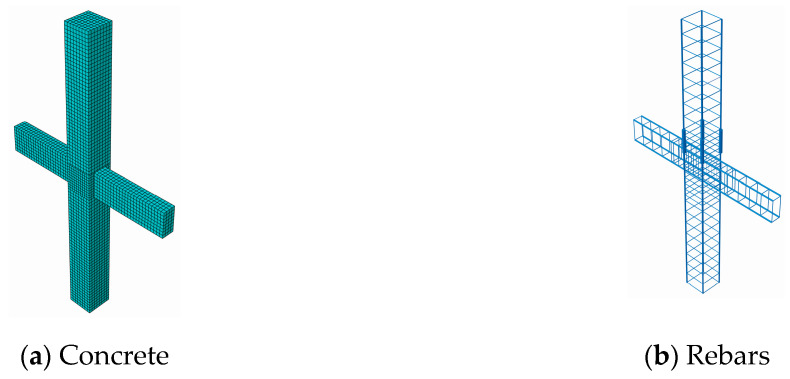
Concrete and rebars of a precast beam–column joint model.

Figure 4 shows the concrete and rebars of a precast beam–column joint model. The joint consisted of a precast column with a cross-section of 400 mm × 400 mm and a height of 4500 mm, and a precast beam with a cross-section of 200 mm × 400 mm and a length of 2800 mm. The concrete compressive strength was 30 MPa, and the tensile strength of the longitudinal reinforcement and stirrup was 400 MPa. Based on the sensitivity study of mesh convergence, an optimal FE mesh that could provide a relatively accurate solution within an acceptable computation time was determined, where the element size of the beam and column was 50 mm, and the element size of the connection was 40 mm.

### 3.2. Working Cases

**Figure 5:**
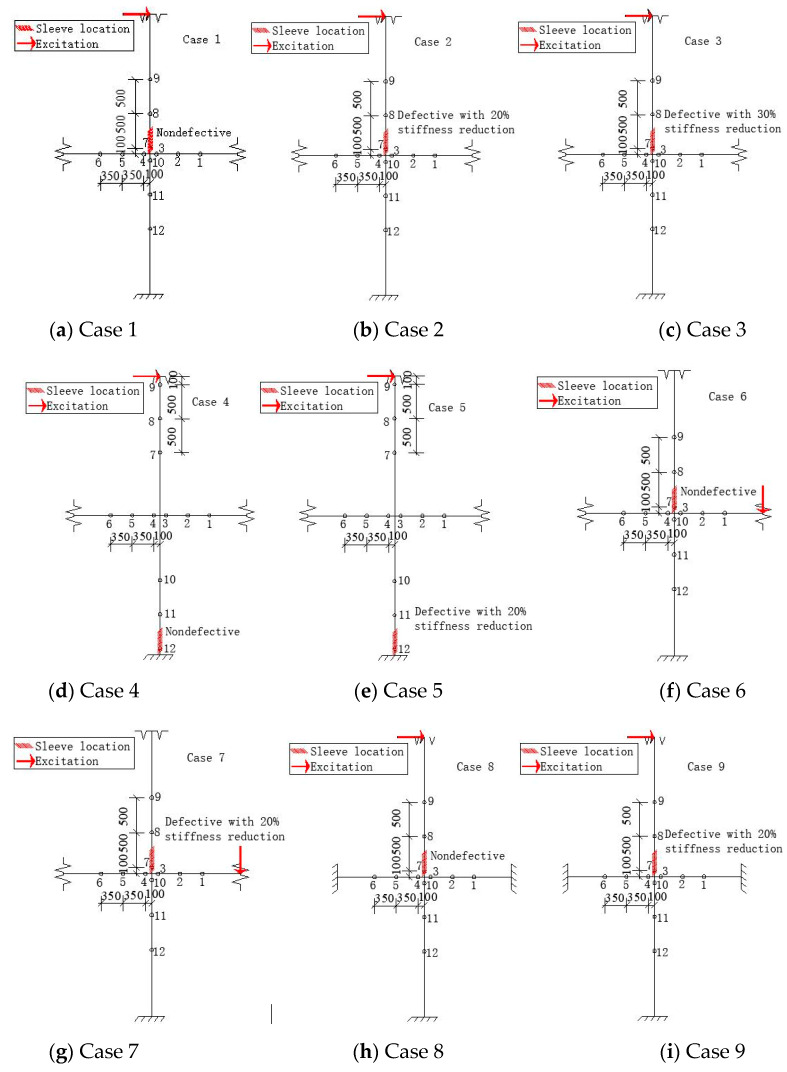
Description of the nine working cases.

To explore the effects of the degree and location of grout defects, the excitation location, and the boundary condition on the algorithm, nine working cases were designed, as shown in Table 1. Figure 5 shows descriptions of these cases. There were 12 measure nodes in each case. Nodes 1–3 were located in the right beam, nodes 4–6 in the left beam, nodes 7–9 in the upper column, and nodes 10–12 in the bottom column. The excitation was harmonic, with a maximum amplitude of 1 kN and frequency of 5 Hz, and acceleration responses at the nodes were collected for 10 s at an interval of 0.05 s.

### 3.3. Results and Discussion

#### 3.3.1. Acceleration Responses

**Figure 6:**
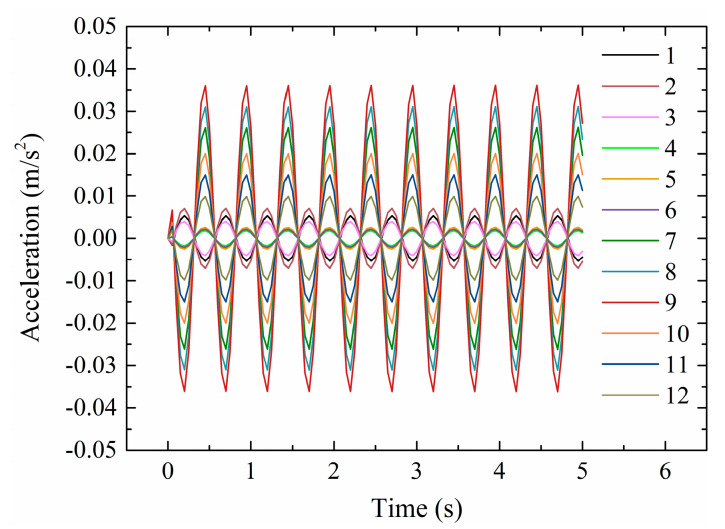
Acceleration response of case 1 in the beam–column connection.

Taking case 1 as an example, the acceleration responses of the 12 nodes are shown in Figure 6 with different maximum amplitudes. 

#### 3.3.2. Results Based on the SVR, TVSR, and TVSTR Models

All acceleration response data from cases 1–9 was analyzed based on the three linear regression models SVR, TVSR, and TVSTR. If the damage indicator values based on different nodes were similar for a case, it was considered that there were no sleeve grout defects in the column. If the damage indicator values of some nodes were significantly larger than others, it was considered that the structural dynamic characteristic near these nodes had changed, which shows that grout defects were located near these nodes. In the experiment, case X_Y represents the analysis based on cases X and Y. For example, “case 1_2” indicates that the damage indicator is calculated according to cases 1 and 2.

1. SVR model

Based on the SVR model, the CoD *r^2^* of every case was calculated. The values of cases 1–5 and 8–9 were almost all equal to 1. The values of cases 6–7 were greater than 0.8, as shown in Table 2. Therefore, the SVR model of every case was reliable.

The damage indicators of cases 1_2 in SVR mode are shown in Table 3. It is seen that the damage indicators of nodes 7–10 were greater than those of other nodes. This shows that for this case, a sleeve defect was near nodes 7–10, which were in the upper column near the beam–column connection. The actual location of the defect was consistent with the identified result. 

To better show the relationship of the damage indicator and damage location, the damage indicators of all pairwise nodes were sorted and summed using:

(9) $$\Delta \theta_{i}=\overset{n}{\underset{j=1}{\sum }}\theta_{ij},$$

where $\Delta \theta_{i}$ is the total damage indicator at node *i*, and $\theta_{ij}$ is the damage indicator of nodes *i* and *j*.

Based on Equation (9), the total damage indicators $\Delta \theta_{i}$ of cases 1_2, 1_3, 4_5, 6_7, and 8_9 at the specified measure nodes were all calculated, as shown in Table 4. It was found that the total damage indicators of nodes 7–10 in cases 1_2 and 1_3 were larger than those of the other nodes, showing that a defect was located in the upper column near node 10. In case 4_5, the first three damage indicator values came from nodes 10–12 located in the bottom column, which means that the defects were located at the positions of nodes 10–12. In case 6_7, the first three values were from nodes 7, 8, and 10, indicating that the defects were near the beam–column joint and upper column. In case 8_9, the first three values were from nodes 7, 8, and 10, indicating that the defects were near the beam–column connection and the upper column. In all cases, the locations of identified defects were in accordance with those of the actual sleeve defects, showing the SVR model was reliable.

2. TVSR model

Based on the TVSR model, the adjusted CoD *r^2^_adj_* of every case was calculated. All the values were nearly equal to 1, which shows that the fitting effect of the estimated regression model was good. 

In the TVSR model, the acceleration responses of the right beam, left beam, upper column, and bottom column in the TVSR model were independently analyzed. The dependent variable data came from nodes 2, 5, 8, and 11. Correspondingly, the responses of nodes 1 and 10, 4 and 6, 7 and 9, and 10 and 12 were respectively taken as the independent variables. The total damage indicators of every case in the TVSR model are shown in Table 5. In cases 1_2 and 1_3, the damage indicator of nodes 7–9 in the upper column was the largest, showing that the defect was located in the upper column. In case 4_5, the total damage indicator of nodes 10–12 was the largest, showing that the defect was located in the bottom column, which was the same as the sleeve defect location. In case 6_7, the total damage indicator of nodes 7–9 was the largest, indicating that the defects were located in the upper column, which was consistent with the location of the grout defect. In case 8_9, the total damage indicator of nodes 10–12 was the largest, indicating the defects were in the bottom column, which was inconsistent with the grout defect location in the upper column. Therefore, the TVSR model algorithm could effectively identify the component where grout defects were located in cases 1_2, 1_3, 4_5, and 6_7, but could not accurately identify the defect in case 8_9.

3. TVSTR model

Based on the TVSTR model, the *r^2^_adj_* of every case was calculated. The values in cases 1, 2, 3 were 1 and in cases 4, 5, 8, and 9, they were almost 1. The adjusted CoDs *r^2^_adj_* of cases 6 and 7 were nearly the same. Taking case 6 as an example, the values of *r^2^_ad_* are shown in Table 6, in which all values were greater than 0.8. Therefore, the TVSTR model was reliable in every case.

Based on the TVSTR model, the total damage indicators in every case were calculated, as shown in Table 7. In cases 1_2 and 1_3, the damage indicators of nodes 7–10 were greater than those of other nodes. It was concluded that the sleeve defect was located in the upper column near node 10. In case 4_5, the damage indicators of nodes 11 and 12 were significantly greater than at other nodes, indicating that the defects were located near nodes 11 and 12. In case 6_7, by comparing the total damage indicators, it was found that the greatest one was node 7, followed by node 8, and the third one was node 10, indicating that the defect was near nodes 7, 8, and 10. In case 8_9, compared with other nodes, the values at nodes 7, 8, and 10 were greater, showing that the defects were near the beam–column connection and the upper column. All the identified results were consistent with the defect design.

#### 3.3.3. Comparison of the SVR, TVSR, and TVSTR Models

**Figure 7:**
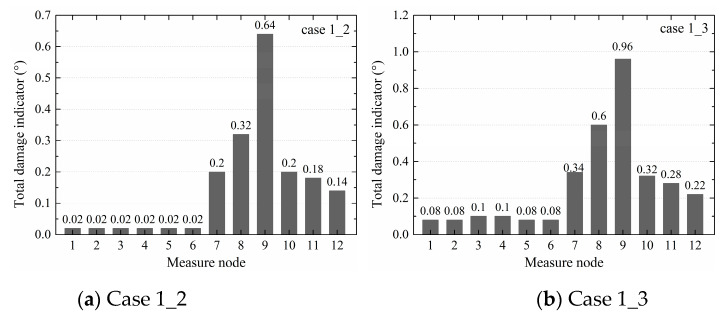
Total damage indicators of cases 1_2 and 1_3 from the SVR models.

**Figure 8:**
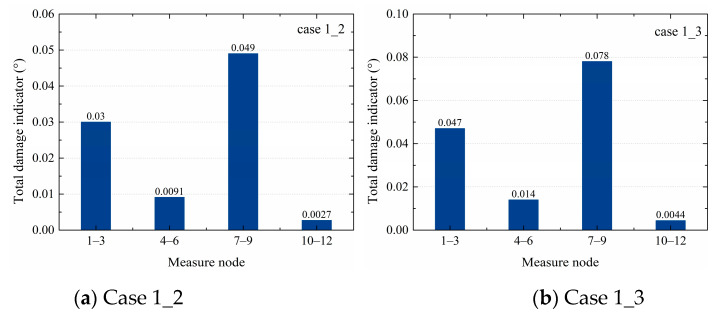
Total damage indicators of cases 1_2 and 1_3 from the TVSR models.

**Figure 9:**
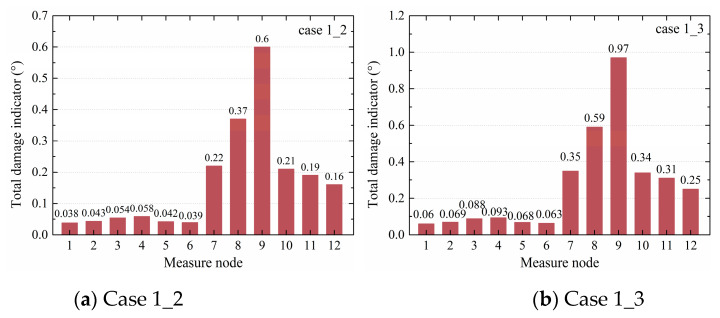
Total damage indicators of cases 1_2 and 1_3 from the TVSTR models.

When comparing the total damage indicators of cases 1_2 and 1_3 from the three models, as shown in Figure 7, Figure 8, and Figure 9, it was noticed that the values of nodes 7–9 in Figure 7b were larger than in Figure 7a, those in Figure 8b were larger than in Figure 8a, and those in Figure 9b were larger than in Figure 9a, indicating that the total damage indicator became larger with the increase of the degree of defects. Furthermore, from all three models, the differences between nodes 7–10 and other nodes in case 1_3 were greater than in case 1_2, showing that the SVR, TVSR, and TVSTR algorithms could more accurately identify components with defects as the degree of defects increased.

**Figure 10:**
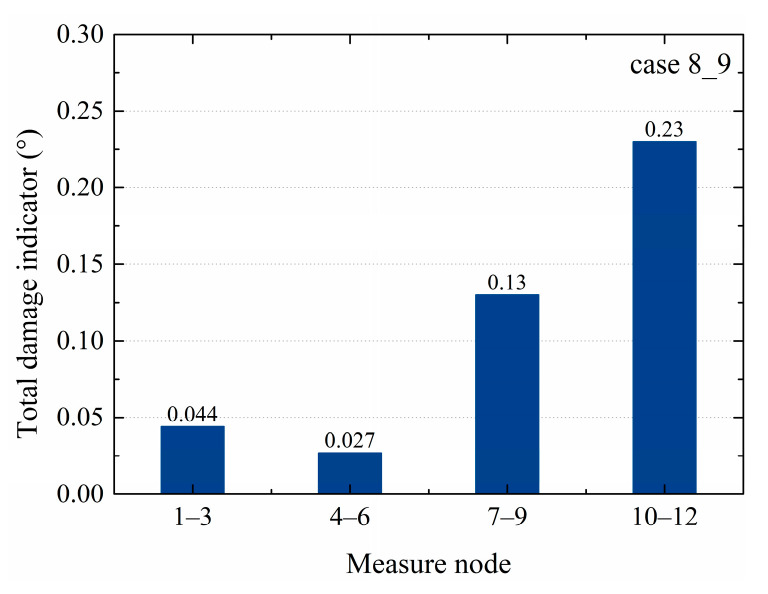
Total damage indicator of case 8_9 from the TVSR model.

For the different cases, the SVR and TVSTR algorithms could identify the structural component where a sleeve grout defect was located well, except for the TVSR model algorithm of case 8_9, as seen in Figure 10, where the nodes 10–12 corresponding to the greatest total damage indicator value were not located near the defect. Additionally, the total damage indicators of nodes 1–3 in Figure 8a,b were relatively large and could not be ignored, which affected the defect identification in a single component. At the same time, the TVSR model algorithm requires that there are at least three measure nodes in each component, where the middle node value is the dependent variable and the other two nodes’ values are the two independent variables. In the precast concrete frame structure, three nodes were designed for each of the right beam, left beam, upper column, and bottom column. The acceleration responses of the middle node were chosen as the dependent variable and the acceleration responses of the other two nodes were chosen as independent variables in the TVSR model. Maybe due to the limited layout and the number of measure nodes, the dependent variable and independent variables were chosen in a limited fashion, which caused the error in case 8_9 and the obscure results in Figure 8a,b. Therefore, the TVSR model algorithm has limitations regarding identifying grout defects in some cases, and based on different layouts and the number of the measure nodes, the application of the TVSR model with different dependent variables and independent variables for unknown defect identification should be further studied.

**Figure 11:**
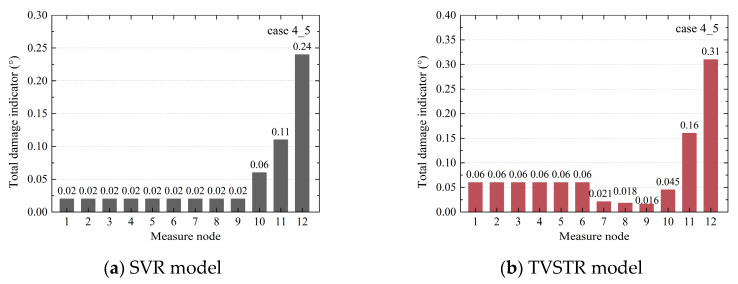
Total damage indicators of case 4_5 from the SVR and TVSTR models.

The total damage indicators of case 4_5 in the SVR and TVSTR models are shown in Figure 11a,b. The value of node 10 seen in Figure 11a was 0.06, and in Figure 11b, it was 0.045, showing the value of node 10 significantly decreased away from the defect in the TVSTR model compared with SVR model. The values of nodes 11 and 12 in Figure 11b were greater than in Figure 11a, indicating that the values of nodes near the defect increased in the TVSTR model compared with the SVR model. In case 6, comparing *r^2^* in the TVR model and *r^2^_adj_* in the TVSTR model, as shown in Table 6 and Table 7, it is seen that all corresponding values were greater in Table 7 than those in Table 6, indicating that the TVSTR model had an improved regression fitting effect than the SVR model. The above shows the TVSTR model could more accurately identify the defect locations in structural components than the SVR model.

#### 3.3.4. Multiple-Variable Regression Model

**Figure 12:**
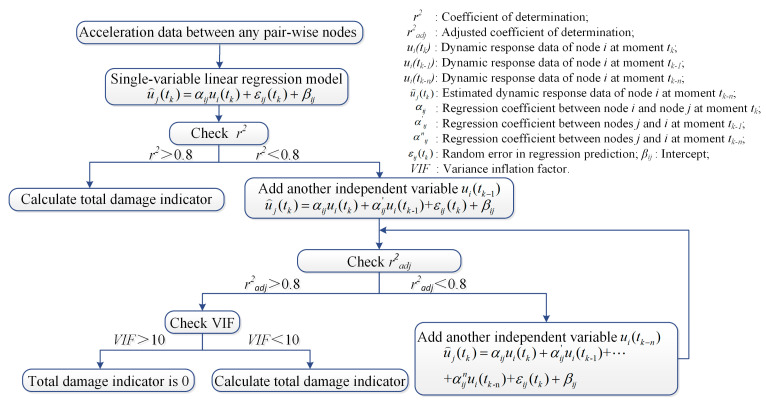
Flowchart of the regression model algorithm for local damage identification.

By analyzing all working cases, it was concluded that the TVSR model considered only the data of adjacent measure nodes and did not fully consider the data of different nodes and moments, resulting in poor defect identification in some cases. Based on the SVR model, an independent variable was added to the TVSTR model to fully consider the linear relationship between the dependent variable and the two independent variables at different moments, contributing to better identification results. Based on the TVSTR model, a multiple-variable spatiotemporal regression model was proposed. Figure 12 shows the integrated process of the regression model recognition algorithm. Independent variables could be continuously added according to the value of *r^2^* or *r^2^_adj_*. A variance inflation factor was used to detect multicollinearity in multiple linear models, where *R^2^* represents *r^2^* or *r^2^_adj_*. If VIF is greater than 10, then it is considered that there is severe collinearity among the independent variables [32,33].

(10) $$VIF=\frac{1}{1-R^{2}}$$

#### 3.3.5. Robustness Analysis of Damage Indicator

Noise inevitably exists in signals obtained in practical engineering. To discuss the ability of the total damage indicator to resist noise and provide justified suggestions for grout defect detection, the robustness analysis was explored. Taking case 1_2 as an example, acceleration signals with signal-to-noise ratios (SNRs) of 1 dB, 5 dB, and 10 dB were analyzed based on a multiple-variable regression model, and the results are shown in Table 8. It is seen that as the level of noise increased, the total damage indicators of nodes 7–10 near the defects became smaller and for nodes 1–6 away from defects, the damage indicators became bigger in general, showing that the identification results tended to be obscured by noise. Meanwhile, for the results with SNRs of 1 dB, 5 dB, and 10 dB inputted, the values of nodes 7–10 were all larger than those of other nodes, indicating a good identification result. In conclusion, although the total damage indicator had a certain robustness against noise with SNRs of 1 dB, 5 dB, and 10 dB inputted, attention needs to paid to avoid the great environmental noise during the experiment to obtain good identification results.

## 4. Experimental Verification of Grout Defect Identification in a Precast Concrete Frame Structure

To verify the effectiveness of the proposed defect identification method, an experiment on a precast concrete frame structure is carried out.

### 4.1. Experimental Model

**Figure 13:**
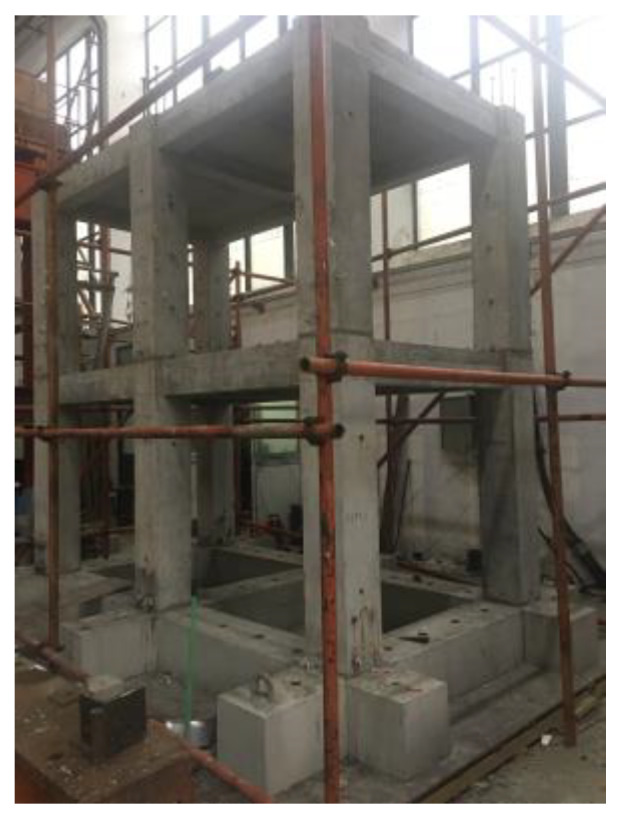
Precast concrete frame structure.

**Figure 14:**
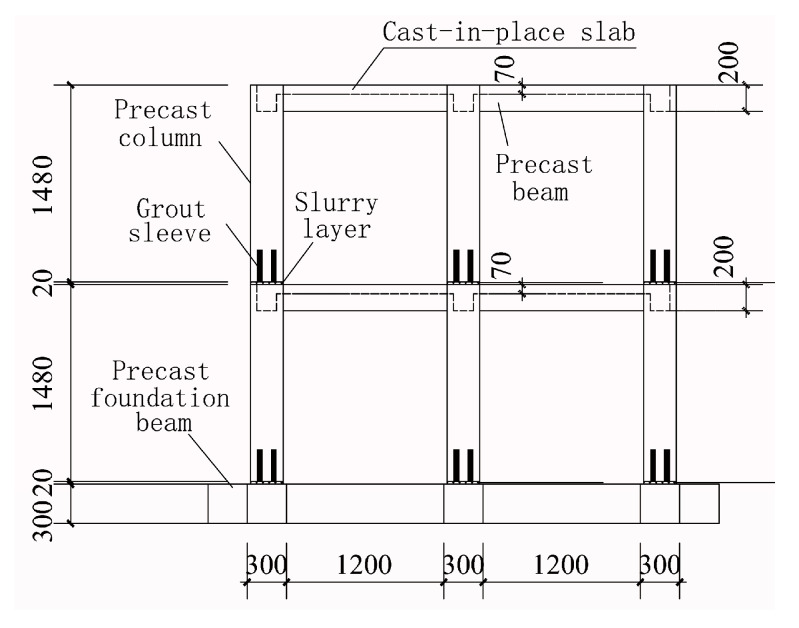
Elevation of the precast concrete frame structure (unit: mm).

A half-scaled, two-floor, precast concrete frame structure with a column space of 1500 mm was constructed, as shown in Figure 13. The structure consisted of precast columns, precast beams, precast foundation beams, cast-in-place slabs, and slurry layers, where all rebars in precast columns were spliced using grout sleeves, as shown in Figure 14. The concrete compressive strength was 30 MPa and the tensile strength of the longitudinal reinforcement and stirrup was 400 MPa.

### 4.2. Experimental Setup

**Figure 15:**
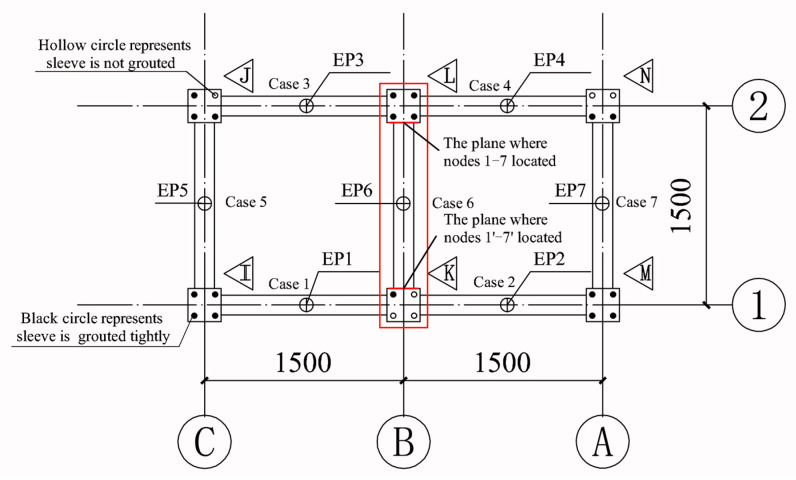
Defects and excitation point (EP) layout of the second floor.

**Figure 16:**
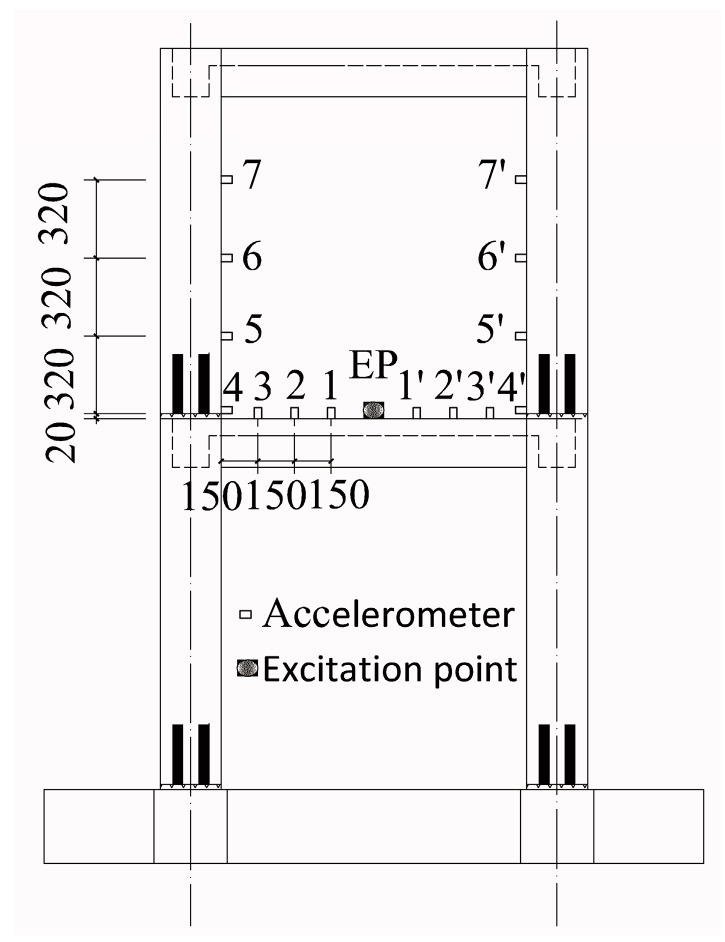
Locations of accelerometers and excitation point in case 6.

For structural safety, the sleeve grout defects were designed to be in the second floor. There were seven cases, as shown in Figure 15. For the convenience of construction, a sleeve with a grout defect was designed without grouting. A hollow circle in a column indicates the sleeve was not grouted completely, and a black circle indicates that it was grouted tightly. It should be noted that in each case, one column was defective and the other was non-defective. To ensure the two columns received the same excitation in each case, the excitation point (EP) was in the middle of the beam, hence there were seven EPs, where EPi indicates an EP in case i. For each case, the locations of the measure nodes were the same. Taking case 6 as an example, the measure nodes arranged along the column and beam are shown in Figure 15 and Figure 16, respectively. There were seven nodes in each column, where nodes 1–3 and 1′–3′ were located at the beam end, and nodes 4–7 and 4′–7′ were located along the column.

**Figure 17:**
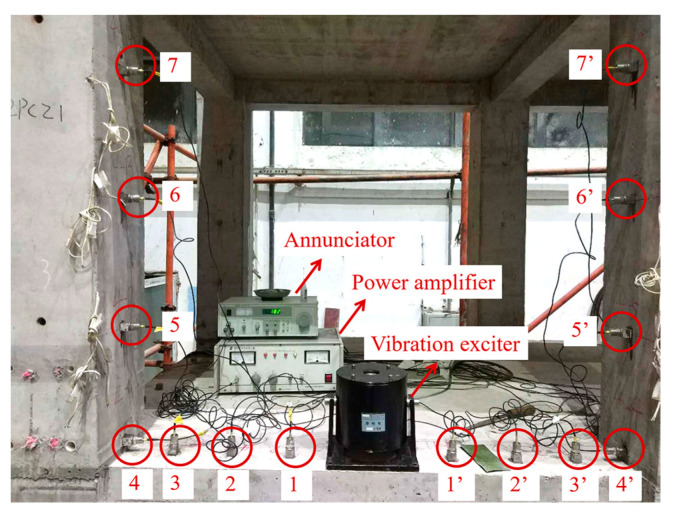
Arrangement of the experimental equipment.

Figure 17 shows the arrangement of the experimental equipment and Table 9 shows the instrument parameters. The acceleration sensors collected the node data, and external excitation was applied using the vibration exciter, signal source, and power amplifier, which were produced by Yangzhou Kedong Electronics Co. LTD in Yangzhuo, China. At the same time, a data acquisition system was used to collect the acceleration responses, which was produced by China Orient Institute of Noise & Vibration in Beijing, China. The excitation force was 200 N and the acquisition frequency was 1024 Hz. 

### 4.3. Experimental Steps

For each case, the experimental steps were the same. Taking case 6 as an example, the experimental steps were as follows:

1. The accelerometers were arranged near the sleeve defects on the columns and beam, whose locations are shown in Figure 16 and Figure 17.
2. The vibration exciter was arranged at the excitation point in the middle of the beam, whose location is shown in Figure 17. At the same time, the annunciator, power amplifier, and acquisition system were arranged as well.
3. Once the external force was applied, the acceleration time history curves of each measuring point were recorded by the acquisition system.
4. Using the proposed multiple-variable regression model, the damage indicators were calculated based on the acceleration responses to identify the defects.

### 4.4. Results and Discussion

#### 4.4.1. Acceleration Responses

**Figure 18:**
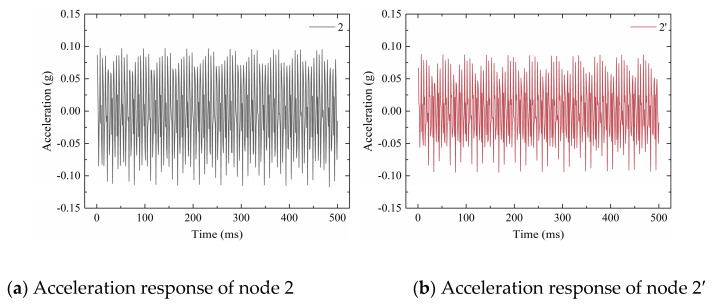
Acceleration responses of nodes 2 and 2’ in case 3.

The acceleration responses of all measuring points in cases 1–7 were similar. Taking nodes 2 and 2′ of case 3 as an example, Figure 18 shows representative portions of the acceleration responses. In the macro view, the responses of nodes 2 and 2′ were different, showing that the grout sleeve defects influenced the structural acceleration responses.

#### 4.4.2. Results Based on Multiple-Variable Regression Model

Cases 1–4 were located in the structural longitudinal direction, and cases 5–7 were in the structural transverse direction. Considering the different boundary conditions, cases 1–4 and cases 5–7 were separately analyzed. Comparing cases 1–4, the defect degrees in cases 1 and 2 were the most severe with three sleeves ungrouted; case 4 was second with two sleeves ungrouted; and in case 3, just one sleeve was ungrouted. Comparing cases 5–7, the defect degree in case 6 was most severe with three sleeves ungrouted, case 7 was second with two sleeves ungrouted, and case 5 had one sleeve ungrouted.

**Figure 19:**
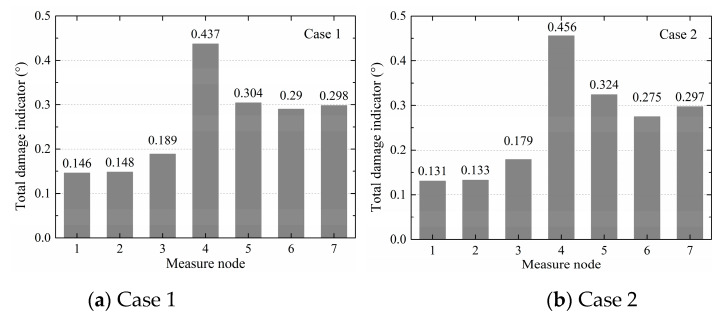

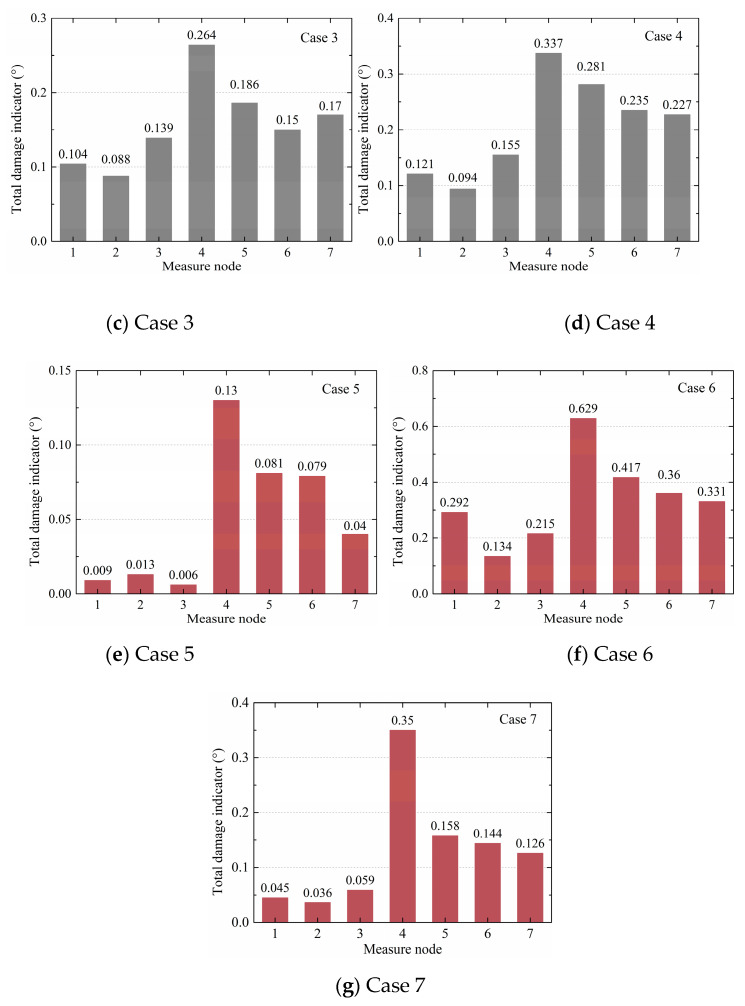
Total damage indicators of cases 1–7 as histograms.

**Figure 20:**
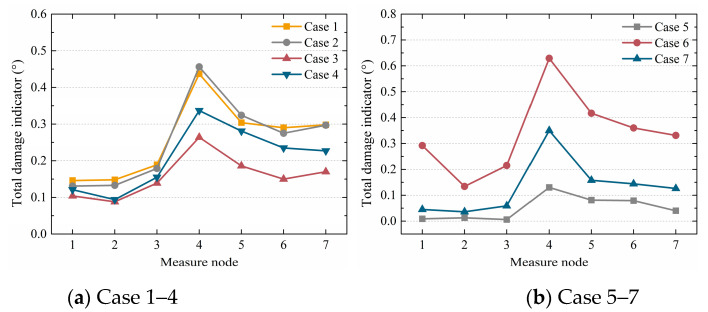
Total damage indicators of cases 1–7 as line charts.

Based on the proposed regression model algorithm for local damage identification in Figure 12, multiple variables were adopted in each case. Figure 19 shows the total damage indicators of cases 1–7 in histogram form. It is seen that the total damage indicators of nodes 4–7 were greater than those of nodes 1–3 for all cases, and the value of node 4 was the greatest. The results indicated that the defect was in the column and near node 4, which was consistent with the defect design. Figure 20 shows the total damage indicators of cases 1–7 in a line chart. Figure 20a shows that the defect degrees in cases 1 and 2 were similar, followed by case 4, and case 3 was last. Considering node 4, the value of case 1 was 0.437; the value of case 2 was close at 0.456; the value of case 4 was 0.337, which was smaller than those of cases 1 and 2; and case 3 had the smallest value of 0.264. Figure 20b shows that the defect degree in case 6 was the most severe, followed by case 7, and the least was that of case 5. Taking node 4 as an example, the value of case 6 was 0.134, the value of case 7 was smaller at 0.036, and the value of case 5 was the least at 0.013. The adjusted CoDs *r^2^_adj_* in all cases were larger than or equal to 0.8. Taking column I in case 5 as an example, the corresponding *r^2^_adj_* are shown in Table 10. Therefore, the proposed regression model algorithm could accurately identify sleeve grout defects in structures.

## 5. Conclusions

This study proposed a grout defect identification method based on a linear regression model algorithm in precast structures, which used the angle between linear regression lines or planes obtained from two measure nodes’ acceleration response as a damage indicator, with the advantage that it was not necessary to know the structural properties in advance. First, a nondestructive vibration test was conducted on precast beam–column joint models simulated in ABAQUS to compare the SVR, TVSR, and TVSTR algorithms, and a multiple-variable regression model was proposed. Based on the model, one nondestructive vibration experiment was conducted on a half-scaled, two-floor, precast concrete frame structure with grout sleeve defects.

The main conclusions were as follows:

1 Comparing the SVR, TVSR, and TVSTR model algorithms, the TVSTR model could most accurately identify the defective components, the SVR was the second best, and the TVSR was the worst, in which the defect of case 8_9 in the numerical simulation could not be identified well. A flowchart of the regression model recognition algorithm was proposed based on multiple spatiotemporal variables.
2 Grout defects in the precast concrete frame structure were successfully identified based on the proposed multiple-variable regression model, with results showing that the total damage indicators of nodes near defects were greater than those of other nodes.
3 The total damage indicator displayed robustness against different levels of noise with SNRs of 1 dB, 5 dB, and 10 dB inputted, but attention still needs to paid to avoid the significant environmental noise that was present during the experiment to obtain good identification results.
4 The proposed method has the limitations that the damage indicator was calculated based on two working conditions, where the structural design and boundary conditions were the same. At the same time, the result could only show the damage difference between the two conditions and one control working case needed to be chosen.

Further research is needed on different layouts and numbers of the measure nodes to put forward efficient suggestions for grout defect identification in precast structure based on a multiple-variable spatiotemporal regression model.

## Figures and Tables

**Table 1:** Working cases in the numerical simulations.

Boundary	Case	Sleeve Location	Degree of Defects	Excitation Location
Fixed supports for the bottom column end, incomplete fixed supports for the other three ends, with no horizontal constraints in the plane	1	Upper column	None	Horizontal rightward on top column end
	2	Upper column	20% stiffness reduction	Horizontal rightward on top column end
	3	Upper column	30% stiffness reduction	Horizontal rightward on top column end
	4	Bottom column	None	Horizontal rightward on top column end
	5	Bottom column	20% stiffness reduction	Horizontal rightward on top column end
	6	Upper column	None	Vertical downward on right beam end
	7	Upper column	20% stiffness reduction	Vertical downward on right beam end
Fixed supports for the four ends	8	Upper column	None	Horizontal rightward on top column end
	9	Upper column	20% stiffness reduction	Horizontal rightward on top column end

**Table 2:** *r^2^* of cases 6–7 in an SVR model.

*r^2^*		Measure Node											
		1	2	3	4	5	6	7	8	9	10	11	12
**Measure Node**	**1**	-	1.00	1.00	1.00	1.00	1.00	0.99	1.00	1.00	1.00	0.81	0.96
	**2**	1.00	-	1.00	1.00	1.00	1.00	0.99	1.00	1.00	1.00	0.81	0.96
	**3**	1.00	1.00	-	1.00	1.00	1.00	1.00	1.00	1.00	1.00	0.81	0.96
	**4**	1.00	1.00	1.00	-	1.00	1.00	0.99	1.00	1.00	1.00	0.82	0.96
	**5**	1.00	1.00	1.00	1.00	-	1.00	0.99	0.99	0.99	1.00	0.82	0.96
	**6**	1.00	1.00	1.00	1.00	1.00	-	0.99	0.99	0.99	0.99	0.83	0.96
	**7**	0.99	0.99	1.00	0.99	0.99	0.99	-	0.99	0.99	0.99	0.84	0.97
	**8**	1.00	1.00	1.00	1.00	0.99	0.99	0.99	-	1.00	1.00	0.80	0.95
	**9**	1.00	1.00	1.00	1.00	0.99	0.99	0.99	1.00	-	1.00	0.80	0.95
	**10**	1.00	1.00	1.00	1.00	1.00	0.99	0.99	1.00	1.00	-	0.81	0.95
	**11**	0.81	0.81	0.81	0.82	0.82	0.83	0.84	0.80	0.80	0.81	-	0.89
	**12**	0.96	0.96	0.96	0.96	0.96	0.96	0.97	0.95	0.95	0.95	0.89	-

**Table 3:** Damage indicator of case 1_2 using the SVR model.

Damage Indicator (°)		Measure Node											
		1	2	3	4	5	6	7	8	9	10	11	12
**Measure Node**	1	-	0.00	0.00	0.00	0.00	0.00	0.00	0.00	0.01	0.00	0.00	0.00
	2	0.00	-	0.00	0.00	0.00	0.00	0.00	0.00	0.01	0.00	0.00	0.00
	3	0.00	0.00	-	0.00	0.00	0.00	0.00	0.00	0.01	0.00	0.00	0.00
	4	0.00	0.00	0.00	-	0.00	0.00	0.00	0.00	0.01	0.00	0.00	0.00
	5	0.00	0.00	0.00	0.00	-	0.00	0.00	0.00	0.01	0.00	0.00	0.00
	6	0.00	0.00	0.00	0.00	0.00	-	0.00	0.00	0.01	0.00	0.00	0.00
	7	0.00	0.00	0.00	0.00	0.00	0.00	-	0.03	0.07	0.00	0.00	0.00
	8	0.00	0.00	0.00	0.00	0.00	0.00	0.03	-	0.03	0.04	0.03	0.03
	9	0.01	0.01	0.01	0.01	0.01	0.01	0.07	0.03	-	0.06	0.06	0.04
	10	0.00	0.00	0.00	0.00	0.00	0.00	0.00	0.04	0.06	-	0.00	0.00
	11	0.00	0.00	0.00	0.00	0.00	0.00	0.00	0.03	0.06	0.00	-	0.00
	12	0.00	0.00	0.00	0.00	0.00	0.00	0.00	0.03	0.04	0.00	0.00	-

**Table 4:** Total damage indicators of every case using the SVR model.

Total Damage Indicator		Measure Node											
		1	2	3	4	5	6	7	8	9	10	11	12
**Case**	**1_2**	0.02	0.02	0.02	0.02	0.02	0.02	0.20	0.32	0.64	0.20	0.18	0.14
	**1_3**	0.08	0.08	0.10	0.10	0.08	0.08	0.34	0.60	0.96	0.32	0.28	0.22
	**4_5**	0.02	0.02	0.02	0.02	0.02	0.02	0.02	0.02	0.02	0.06	0.11	0.24
	**6_7**	0.02	0.02	0.02	0.03	0.04	0.06	0.22	0.12	0.10	0.19	0.09	0.09
	**8_9**	0.55	0.58	0.61	0.47	0.49	0.49	1.09	1.24	0.90	1.24	0.94	0.84

**Table 5:** Total damage indicators of every case using the TVSR model.

Total Damage Indicator		Measure Node			
		1–3	4–6	7–9	10–12
**Case**	**1_2**	0.030	0.0091	0.049	0.0027
	**1_3**	0.047	0.014	0.078	0.0044
	**4_5**	0.0021	0.0022	0	0.12
	**6_7**	0.011	0.0077	0.061	0.0019
	**8_9**	0.044	0.027	0.13	0.23

**Table 6:** *r^2^_adj_* of case 6 in a TVSTR model.

*r^2^_adj_*		Measure Node											
		1	2	3	4	5	6	7	8	9	10	11	12
**Measure Node**	**1**	-	1.00	1.00	1.00	1.00	1.00	1.00	1.00	1.00	1.00	0.84	0.98
	**2**	1.00	-	1.00	1.00	1.00	1.00	1.00	1.00	1.00	1.00	0.84	0.98
	**3**	1.00	1.00	-	1.00	1.00	1.00	1.00	1.00	1.00	1.00	0.84	0.98
	**4**	1.00	1.00	1.00	-	1.00	1.00	0.99	1.00	1.00	1.00	0.85	0.98
	**5**	1.00	1.00	1.00	1.00	-	1.00	0.99	0.99	0.99	1.00	0.85	0.98
	**6**	1.00	1.00	1.00	1.00	1.00	-	0.99	0.99	0.99	0.99	0.86	0.98
	**7**	0.99	0.99	1.00	0.99	0.99	0.99	-	0.99	0.99	0.99	0.86	0.99
	**8**	1.00	1.00	1.00	1.00	0.99	0.99	0.99	-	1.00	1.00	0.83	0.97
	**9**	1.00	1.00	1.00	1.00	0.99	0.99	0.99	1.00	-	1.00	0.83	0.97
	**10**	1.00	1.00	1.00	1.00	1.00	0.99	0.99	1.00	1.00	-	0.83	0.97
	**11**	0.87	0.87	0.87	0.87	0.88	0.88	0.89	0.85	0.85	0.85	-	0.93
	**12**	0.98	0.98	0.98	0.98	0.98	0.98	0.99	0.97	0.97	0.97	0.91	-

**Table 7:** Total damage indicators of every case using the TVSTR model.

Total Damage Indicator		Measure Node											
		1	2	3	4	5	6	7	8	9	10	11	12
**Case**	**1_2**	0.038	0.043	0.054	0.058	0.042	0.039	0.22	0.37	0.60	0.21	0.19	0.16
	**1_3**	0.060	0.069	0.088	0.093	0.068	0.063	0.35	0.59	0.97	0.34	0.31	0.25
	**4_5**	0.060	0.060	0.060	0.060	0.060	0.060	0.021	0.018	0.016	0.045	0.16	0.31
	**6_7**	0.047	0.052	0.064	0.054	0.066	0.085	0.24	0.19	0.15	0.17	0.12	0.075
	**8_9**	0.54	0.57	0.61	0.47	0.50	0.46	1.15	1.23	0.86	1.17	0.86	0.76

**Table 8:** Results comparison based on signals with different levels of noise.

Total Damage Indicator		Measure Node											
		1	2	3	4	5	6	7	8	9	10	11	12
**Signal-to-Noise** **Ratio**	**0 dB**	0.02	0.02	0.02	0.02	0.02	0.02	0.20	0.32	0.64	0.20	0.18	0.14
	**1 dB**	0.02	0.021	0.03	0.025	0.03	0.03	0.20	0.31	0.64	0.19	0.18	0.15
	**5 dB**	0.03	0.04	0.042	0.044	0.043	0.046	0.19	0.29	0.60	0.19	0.17	0.15
	**10 dB**	0.042	0.06	0.058	0.064	0.065	0.064	0.18	0.25	0.58	0.18	0.17	0.16

**Table 9:** Instrument parameters.

Instrument	Model	Overview	Features
Signal source	KD5602	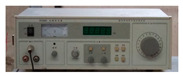	Output mode: Sinusoidal, logarithmic, linear Frequency range: 10 Hz–20 kHz
Power amplifier	KD5702	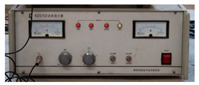	Rated output power: 200 W Rated output voltage: 14 V Rated output current: 15 A Frequency range: 20 Hz–10 kHz
Vibration exciter	KDJ-20	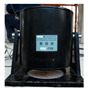	Maximum force: 200 N Maximum amplitude: ±5 mm Frequency range: DC ≈2 kHz
Acceleration sensor	KD8-LP16D	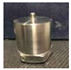	Calibration value: about 60 mV/g
Data acquisition system	INV3060	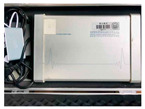	Corresponding acquisition software: DASP-V10 produced by China Orient Institute of Noise & Vibration

**Table 10:** *r^2^_adj_* of column I in case 5.

*r^2^_adj_*		Measure Node						
		1	2	3	4	5	6	7
**Measure Node**	**1**	1.00	0.95	0.88	0.83	0.80	0.81	0.82
	**2**	0.95	1.00	0.92	0.87	0.82	0.97	0.83
	**3**	0.87	0.91	1.00	0.84	0.86	0.91	0.89
	**4**	0.83	0.86	0.83	1.00	0.84	0.80	0.83
	**5**	0.80	0.82	0.86	0.85	1.00	0.88	0.95
	**6**	0.80	0.96	0.91	0.81	0.87	1.00	0.89
	**7**	0.82	0.83	0.89	0.84	0.95	0.90	1.00
